# Cognitive Impairment, Sleep Disturbance, and Depression in Women with Silicone Breast Implants: Association with Autoantibodies against Autonomic Nervous System Receptors

**DOI:** 10.3390/biom12060776

**Published:** 2022-06-02

**Authors:** Milena Tocut, Gilad Halpert, Avishai M. Tsur, Kassem Sharif, Harald Heidecke, Yair Levy, Abdulla Watad, Howard Amital, Yehuda Shoenfeld

**Affiliations:** 1 Department of Medicine C, Wolfson Medical Center, Affiliated to Sackler Faculty of Medicine, Tel Aviv University, Tel Aviv 69978, Israel; milena.tocut@gmail.com; 2 Zabludowicz Center for Autoimmune Diseases, Sheba Medical Center, Tel-Hashomer, Ramat Gan 52621, Affiliated to Sackler Faculty of Medicine, Tel Aviv University, Tel Aviv 69978, Israel; yehuda.shoenfeld@sheba.health.gov.il; 3 Department of Medicine ‘B’ and Zabludowicz Center for Autoimmune Diseases, Sheba Medical Center, Tel-Hashomer, Ramat Gan 52621, Affiliated to Sackler Faculty of Medicine, Tel Aviv University, Tel Aviv 69978, Israel; avishaitsur@gmail.com (A.M.T.); kassem.sharif7001@gmail.com (K.S.); watad.abdulla@gmail.com (A.W.); howard.amital@sheba.health.gov.il (H.A.); 4 Israel Defense Forces, Medical Corps, Tel-Hashomer, Ramat Gan, Affiliated with the Department of Military Medicine, Hebrew University of Jerusalem Faculty of Medicine, Jerusalem 9112102, Israel; 5 CellTrend GmbH, 14943 Luckenwalde, Germany; heidecke@celltrend.de; 6 Department of Medicine E, Meir Medical Center, Kfar Saba 4428164, Affiliated to Sackler Faculty of Medicine, Tel Aviv University, Tel Aviv 69978, Israel; levy.yair@clalit.org.il; 7 Ariel University, Ariel 4077625, Israel

**Keywords:** silicone breast implants, α and β adrenergic receptors, muscarinic acetylcholine receptors, endothelin receptor type A, type 1 angiotensin II receptor, autoantibodies

## Abstract

Background: Silicone breast implants (SBIs) has been shown to be associated with an increased risk of autoimmune diseases. In the current study, we aimed to explore the potential association between circulating autoantibodies against the autonomic nervous system and cognitive impairment, memory deficit, and depressive symptoms reported by women with SBIs. Methods: ELISA assays were used to quantify anti-adrenergic receptors (α1, α2, β1, β2), anti-muscarinic receptors (M1-M5), anti-endothelin receptor type A, and anti-angiotensin II type 1 receptor titers in the sera of 93 symptomatic female subjects with SBIs and 36 age-matched healthy female controls. Results: A significant difference was detected in the level of autoantibodies against the autonomic nervous system receptors in women with SBIs who reported memory impairment, cognitive impairment, and sleep disturbance as compared with both women with SBIs who did not complain of these symptoms or with healthy individuals without SBIs. Conclusions: Clinical symptoms such as depression, cognitive impairment, and sleep disturbances were found to be associated with dysregulation of the levels of circulating autoantibodies targeting the autonomous nervous system receptors in women with SBIs. These autoantibodies may have diagnostic significance in diseases associated with breast implants.

## 1. Introduction

Silicone breast implants (SBIs) are medically approved for use in either breast reconstruction after breast cancer mastectomy or for augmentation purposes. SBIs are associated with autoimmune phenomena, in both intact silicone implants as well as in ruptured implants secondary to either local silicone seepage, or distant silicone gel migration; however, causal evidence is still lacking [1,2,3]. In genetically predisposed individuals, silicone acts as an adjuvant and results in the hyperstimulation of the host immune system [4,5]. The involvement of the adaptive immune system in the autoimmune/inflammatory syndrome induced by adjuvants (ASIA) is well-established [6]. As a result, various autoimmune phenomena have been identified such as fibromyalgia and undifferentiated connective tissue diseases [7], and the hyperstimulated adaptive immunity could result in non-Hodgkin lymphomas [5]. Our group previously reported the presence of several autoantibodies including serum amyloid A (SSA), serum amyloid B (SSB), histone ribosomal phosphate, Scl-70, cardiolipin, phosphatidylserine, GM2-ganglioside, and NC-1 in symptomatic women with SBIs, as well as a significant association between SBIs and Sjogren’s syndrome, systemic sclerosis, and sarcoidosis in a large epidemiological study [8,9]. The immunopathogenesis of entities such as Sjogren’s syndrome, sarcoidosis, and undifferentiated connective tissue diseases are not well-understood; however, they are shown to share several of the common pathogenic aspects of ASIA. Patients with Sjogren’s syndrome, sarcoidosis, and undifferentiated connective tissue diseases have been shown to oftentimes fulfill the diagnostic criteria of ASIA [10].

The discovery of functional autoantibodies resulted in a paradigm shift in our understanding of both the agnostic and antagonistic physiologic pathways in the autonomous central nervous system [11]. Such autoantibodies target G-protein-coupled receptors (GPCRs), the predominant integral cell membrane proteins in the immune and non-immune cells, and interfere with intracellular signaling pathways, resulting in disturbance of body homeostasis and the subsequent emergence of autoimmune conditions including Sjogren’s syndrome, rheumatoid arthritis, systemic sclerosis, etc. [12,13,14,15].

Several functional immunoglobulin G (IgG) autoantibodies targeting GPCRs are associated with autoimmune diseases, including anti-adrenergic receptors (α1AR, α2AR, β1AR, and β2AR), anti-muscarinic acetylcholine receptors (M1R–M5R), anti-endothelin receptor type A (ETAR), and anti-type 1 angiotensin II receptor (AT1R) [16]. An association between those autoantibodies and cardiovascular diseases (hypertension, cardiomyopathies, congestive heart failure) [17], respiratory diseases (asthma, no smoking lung emphysema) [18], autoimmune diseases (systemic lupus erythematosus, rheumatoid arthritis, systemic sclerosis) [16], and myalgic encephalomyelitis/chronic fatigue syndrome (ME/CFS) was previously reported [19]. Despite the high titers of adrenergic β1AR and β2AR and muscarinic M3R and M4R autoantibodies detected in the cerebrospinal fluid in patients with ME/CFS, the functional activity of such autoantibodies remains unaddressed [20]. Interestingly, anti-neuronal antibodies targeting the central and enteric nervous system were found to contribute to the extraintestinal neurological manifestations of coeliac disease, an immune-mediated gastrointestinal condition with female preponderance [21].

Accumulating lines of evidence highlight the role of GPCR-mediated noradrenergic secretion on cognition. The α2AR receptor has a major role in the noradrenergic transmission cascade in depressive disorders [17,22], memory impairment [23,24], and Alzheimer’s disease [25,26]. Moreover, cholinergic neurotransmission via the muscarinic receptors located in the hippocampus and amygdala has been associated with memory impairment and Alzheimer’s disease [27,28].

In a recent publication, our group demonstrated dysregulation of the level of circulating autoantibodies against autonomic nervous system GPCRs in symptomatic women with SBIs suffering from subjective and autonomic-related manifestations such as palpitations, extensive pain, depression, hearing loss, and dry eyes and mouth [29].

In the current study, we set to investigate the association between circulating autoantibodies to adrenergic, muscarinic, endothelin receptor type A (ETA), and AT1 receptors and specific clinical manifestations of depression, cognitive impairment including memory disorders, and sleep disturbances in symptomatic women with SBIs.

## 2. Material and Methods

### 2.1. Study Design

We conducted a cross-sectional, single-center study. The study was approved by the institutional review board of the Sheba Medical Center, according to the Declaration of Helsinki (approval no: 6619-19-MSC; approval date: 4 March 2020). The patients signed a written, informed consent form. The manuscript was written according to the Strengthening the reporting of observational studies in epidemiology (STROBE) statement [30].

### 2.2. Patient Recruitment

Subjects attended the Zabludowicz Center for Autoimmune Diseases, Sheba Medical Center, Israel, due to diverse ill-defined symptoms and clinical manifestations that were believed to be related to SBIs. Inclusion criteria entailed the subject’s reported symptoms and SBI primary indication (augmentation or reconstruction). Exclusion criteria included the history of SBI removal. The study included 93 symptomatic female subjects with SBIs. The median age was 41 years, interquartile range (IQR) of 35–49. The median time from silicone breast implantation to the onset of symptoms was 11.0 years (6.0–14.0). Among the 93 women with SBIs, 19 underwent implant for reconstruction purposes (20.4%) while 74 underwent breast implantation for cosmetic purposes (79.6%). An extensive, structured interview conducted by a rheumatologist/immunologist was used to collect clinical data such as a past medical history of autoimmune diseases, familial history of autoimmune diseases, and the time period between SBI implantation and symptoms onset. The demographics of the enrolled participants were presented in our previous published study [12]. The control group included 36 age-matched healthy females that were chosen from the Magen David Adom, Israel’s National Emergency Pre-Hospital Medical and Blood Services Organization. The median age of healthy donors was 41 years, with an IQR of 35–49.

### 2.3. Quantification of Circulating Autoantibody Levels

Whole-blood samples were withdrawn in order to quantify circulating autoantibodies titers for the anti-adrenergic receptors (α1, α2, β1, β2), anti-muscarinic receptors (M1-M5), anti-endothelin receptor type A (ETAR), and anti-angiotensin II type 1 receptor (AT1R). The median time of blood withdrawal in symptomatic SBI subjects was 11 years post-implantation. Blood was clotted at room temperature and then centrifuged at 2000× *g* for 15 min in a refrigerated centrifuge. Sera were purified and stored at −35 °C. The circulating autoantibody titers were measured in the serum samples using a sandwich ELISA kit (CellTrend GmbH Luckenwalde, Germany). The microtiter 96-well polystyrene plates were coated with GPCR. To maintain the conformational epitopes of the receptor, 1 mM calcium chloride was added to every buffer. Duplicated samples of a 1:100 serum dilution were stored at 4 °C for 2 h. After washing steps, plates were kept for 60 min with a 1:20,000 dilution of horseradish-peroxidase-labeled goat anti-human IgG used for detection. In order to obtain a standard curve, plates were subjected to test serum from an anti-GPCR autoantibody-positive index patient. The ELISAs were validated according to the FDA’s “Guidance for industry: Bioanalytical method validation”. The optimal cut-off level for each anti-GPCR autoantibody test was analyzed using the receiver operating characteristic (ROC) analysis, as described previously.

### 2.4. Statistical Analysis

Continuous variables were presented as median (IQR) and compared using Mann–Whitney U test. *p*-values were adjusted for multiple comparisons, and *p* < 0.05 was considered statistically significant. Data analysis was performed using R version 4.0.4 (R Core Team, Vienna, Austria).

## 3. Results

Recruited subjects were classified into one of three groups—namely, healthy controls without SBIs; symptomatic subjects with SBIs who reported memory disorders, sleep disturbances, cognitive impairment, and/or depression; and subjects with SBIs who did not report such symptoms.

Within the studied group of 93 women with SBIs, 53 patients (57%) experienced memory disorders, 52 patients (56%) complained of sleep disturbances, 42 patients (45%) endured cognitive impairment, and 37 patients (40%) had depression. Notably, the rest of the women with SBIs did not suffer from such symptoms (memory impairment, cognitive impairment, sleep disturbance) but possibly suffered from other symptoms, as previously described by us (29).

**Figure 1:**
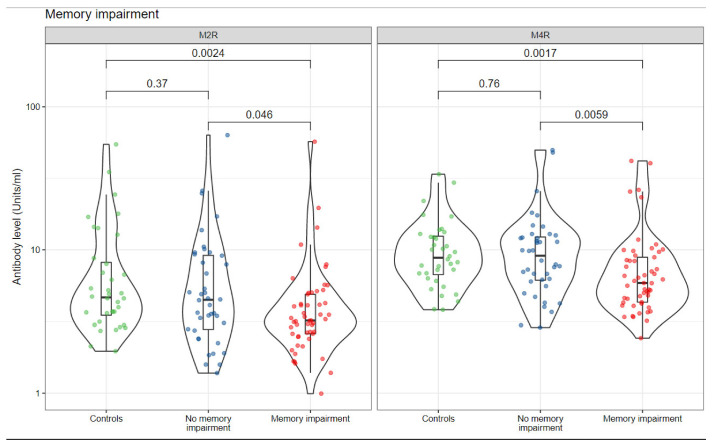
Autoantibodies against autonomic nervous system receptors are correlated with memory impairment in symptomatic women with SBIs. Individual measurements are shown as dots, summary data as box plots, and the distributions as violin plots. Green dots: healthy controls; Blue dots: silicone-breast-implant patients without clinical manifestations; red dots: silicone-breast-implant patients with clinical manifestations.

First, we examined the potential association between the circulating level of autoantibodies against the autonomic nervous system receptors with memory disorders in women with SBIs. It was found that SBI patients who reported memory disorders had significantly lower median titers of anti-M2R (3 vs. 4, *p* = 0.046) and anti-M4R (6 vs. 9, *p* = 0.006) in comparison to SBI patients who did not report memory disorders (Figure 1 and Table 1). Notably, SBI patients who reported memory disorders had significantly lower median titers of anti-M2R (3 vs. 5, *p* = 0.002) and anti-M4R (6 vs. 9, *p* = 0.001) in comparison to the healthy control group. Moreover, no statistically significant difference was detected in the median titers of anti-M2R (*p* = 0.37) and anti-M4R (*p* = 0.76) autoantibodies when comparing the healthy control group without SBIs to SBI subjects who did not suffer from memory disorders (Figure 1 and Table 1).

**Figure 2:**
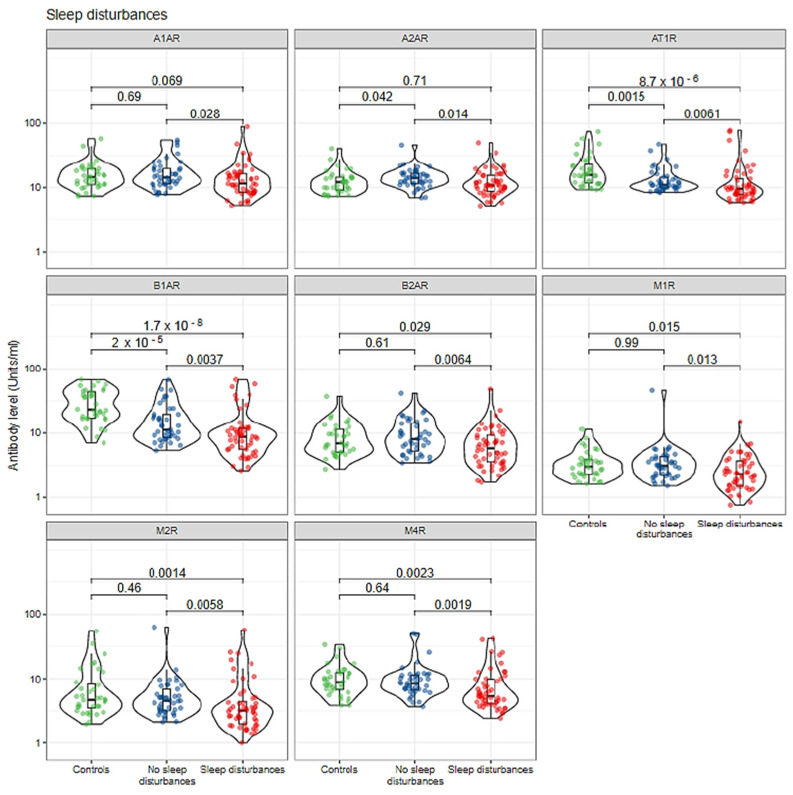
Autoantibodies against autonomic nervous system receptors are correlated with sleep disturbance in symptomatic women with SBIs. Individual measurements are shown as dots, summary data as box plots, and the distributions as violin plots. Green dots: healthy controls; Blue dots: silicone-breast-implant patients without clinical manifestations; red dots: silicone-breast-implant patients with clinical manifestations.

Exploring the potential association between circulating level of autoantibodies against the autonomic nervous system receptors with sleep disturbance in women with SBIs, it was found that SBI patients who suffered from sleep disturbance had significantly lower median titers of anti-α1AR (12 vs. 14, *p* = 0.028), anti-α2AR (10.9 vs. 14.1, *p* = 0.014), anti-AT1R (10 vs. 11, *p* = 0.006), anti-β1AR (9 vs. 11, *p* = 0.003), anti-β2AR (5.8 vs. 8.1, *p* = 0.006), anti-M1R (2.31 vs. 3.12, *p* = 0.013), anti-M2R (3 vs. 5, *p* = 0.005), and anti-M4R (5 vs. 8, *p* = 0.001) in comparison to SBI patients who did not suffer from sleep disturbance (Figure 2 and Table 2). Notably, SBI patients who reported memory disorders had significantly lower median titers of anti-M2R (3 vs. 5, *p* = 0.002) and anti-M4R (5 vs. 9, *p* = 0.001) in comparison to the healthy control group. Moreover, there was no significant difference in the level of anti-α1AR (*p* = 0.69), anti-β2AR (*p* = 0.61), anti-M1R (*p* = 0.99), anti-M2R (*p* = 0.46), and anti-M4R (*p* = 0.64) autoantibodies when comparing the healthy control group without SBIs to subjects with SBI subjects who did not suffer from sleep disturbance (Figure 2 and Table 2). Interestingly, only anti-AT1R and anti-β1AR antibodies were found to be significantly different amongst all three studied groups (Figure 2 and Table 2).

**Figure 3:**
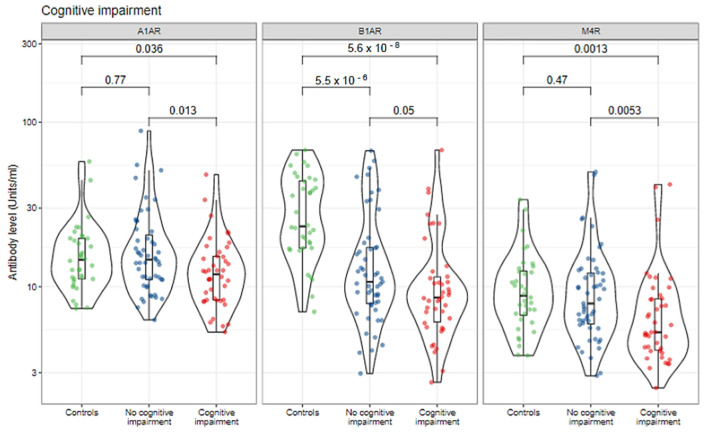
Autoantibodies against autonomic nervous system receptors are correlated with cognitive impairment in symptomatic women with SBIs. Individual measurements are shown as dots, summary data as box plots, and the distributions as violin plots. Green dots: healthy controls; blue dots: silicone-breast-implant patients without clinical manifestations; red dots: silicone-breast-implant patients with clinical manifestations.

When analyzing the association between circulating level of autoantibodies with cognitive impairment, SBI patients who reported cognitive impairment had significantly lower median titers of anti-α1AR (12 vs. 15, *p* = 0.013), anti-β1AR (9 vs. 11, *p* = 0.05), and anti-M4R (5 vs. 8, *p* = 0.005) in comparison to SBI patients who did not report cognitive impairment (Figure 3 and Table 3). Notably, SBI patients who reported cognitive impairment had significantly lower median titers of anti-α1AR (12 vs. 15, *p* = 0.036), anti-β1AR (9 vs. 23, *p* < 0.001), and anti-M4R (5 vs. 9, *p* = 0.001) in comparison to healthy controls without SBIs. Moreover, there was no significant difference in the median titers of anti-α1AR (*p* = 0.77) and anti-M4R (*p* = 0.47) autoantibodies when comparing healthy controls without SBIs to subjects with SBIs who did not report cognitive impairment (Figure 3 and Table 3). Further, only anti-β1AR antibodies were found to be significantly different between all three groups (Figure 3 and Table 3).

**Figure 4:**
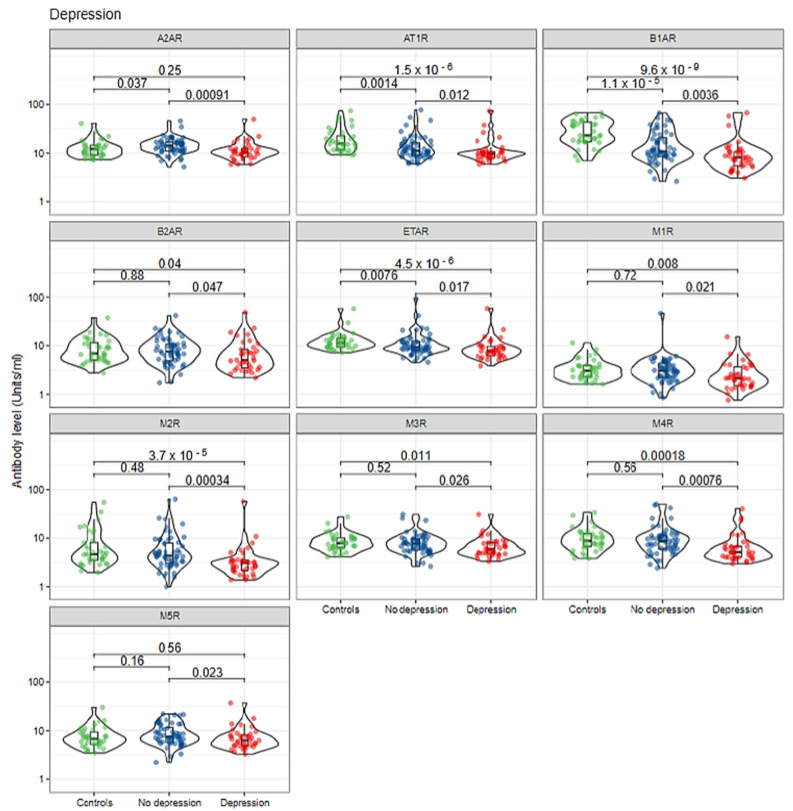
Autoantibodies against autonomic nervous system receptors are correlated with depression in symptomatic women with SBIs. Individual measurements are shown as dots, summary data as box plots, and the distributions as violin plots. Green dots: healthy controls; blue dots: silicone-breast-implant patients without clinical manifestations; red dots: silicone-breast-implant patients with clinical manifestations.

Lastly, SBI patients who reported depression had significantly lower median titers of anti-α2AR (10.3 vs. 14.2, *p* < 0.001), anti-AT1R (10 vs. 11, *p* = 0.012), anti-β1AR (8 vs. 11, *p* = 0.003), anti-β2AR (5.1 vs. 7.5, *p* = 0.047), anti-ETAR (7.7 vs. 9.3, *p* = 0.017), anti-M1R (2.13 vs. 3.05, *p* = 0.021), anti-M2R (3 vs. 4, *p* < 0.001), anti-M3R (5.9 vs. 7.7, *p* = 0.026), anti-M4R (5 vs. 9, *p* < 0.001), and anti-M5R (6.3 vs. 7.6, *p* = 0.023) in comparison to SBI patients who did not report depression (Figure 4 and Table 4). SBI patients who reported depression had significantly lower median titers of anti-AT1R (10 vs. 16, *p* < 0.001), anti-β1AR (8 vs. 23, *p* < 0.001), anti-β2AR (5.1 vs. 6.9, *p* = 0.04), anti-ETAR (7.7 vs. 11.3, *p* < 0.001), anti-M1R (2.13 vs. 3.04, *p* = 0.008), anti-M2R (3 vs. 5, *p* < 0.001), anti-M3R (5.9 vs. 7.9, *p* = 0.011) and anti-M4R (5 vs. 9, *p* < 0.001) in comparison to healthy controls without SBIs. No significant differences were detected in the median titers of anti-β2AR (*p* = 0.88), anti-M1R (*p* = 0.72), anti-M2R (*p* = 0.48), anti-M3R (*p* = 0.52), anti-M4R (*p* = 0.56) and anti-M5R (*p* = 0.16) autoantibodies between healthy controls without SBIs and subjects with SBIs who did not report depression (Figure 4 and Table 4). Interestingly, anti-AT1R, anti-β1AR, and anti-ETAR antibodies were found to be significantly different between all three groups (Figure 4 and Table 4).

## 4. Discussion

Based on our reported results, we propose that depression, cognitive impairment including memory deficits, and sleep disturbances in symptomatic women with SBIs are associated with dysregulation of the circulating levels of functional autoantibodies targeting the autonomic nervous system receptors such as α and β adrenergic receptors, muscarinic acetylcholine receptors, endothelin receptor type A, and type 1 angiotensin II receptors.

It is suggested that autoantibodies targeting neuronal surface antigens such as neuronal ion channels or neurotransmitter receptors result in severe functional disorders such as schizophrenia, bipolar disorders, depression, and dementia [31,32]. Earlier reports on autonomic central nervous system receptor autoantibodies including N-methyl-d-aspartate-receptor subunit NR1 (NMDAR1), α-amino-3-hydroxy-5-methyl-4-isoxazolepropionic acid receptor (AMPAR), GABAB receptor (GABABR) and dipeptidyl aminopeptidase-like protein 6 (DPPX), were described in autoimmune encephalitis [33,34]. Cognitive and memory dysfunction in neurodegenerative diseases such as Alzheimer’s disease is postulated to be attributed to increasing titers of serum autoantibodies against 5-hydroxytryptamine receptors (5-HT2AR, 5-HT2CR, and 5-HT7R), vascular endothelial growth receptor 1 (VEGFR1), Stabilin-1 (Stab1), NMDAR, and endothelin type A receptors (ETAR). The existence of the mentioned receptors is also associated with a high mortality rate in patients with Alzheimer’s [31,35].

Muscarinic and adrenergic receptors play crucial roles in learning and memory, most prominently M1R [27,28,36] and β1AR [37]. It was suggested that autoantibodies to adrenergic receptor β1AR and muscarinic receptors M1-M3R were associated with mood disorders in patients with Alzheimer’s disease [35]. To date, increasing evidence points toward the role of autoantibodies to the adrenergic receptors α1AR and β1AR in the pathogenesis of vascular dementia and Alzheimer’s disease [38,39] with autoantibodies detected in at least 59% of patients [40]. Other reports demonstrated a causal relationship between α1AR and β1AR autoantibodies dementia; however, no correlation with severity was established [41].

In this report, we report cognitive impairment and memory dysfunction in women with SBIs, which was associated with measured serum titers of receptor autoantibodies to α1AR, β1AR, M2R, and M4R. Interestingly, our findings indicate a reduction in levels of these autoantibodies in patients suffering from cognitive impairment and memory disorders as opposed to increased levels reported in previous studies [38,39,40,41]. The reason for this discrepancy is not fully understood; however, recent studies showed a reduction in the level of specific anti-GPCRs autoantibodies, including anti-β1AR and anti-ETAR autoantibodies, in the sera of patients with autoimmune diseases, and acute coronary syndrome, compared with healthy donors [13,42], potentially attributed to autoantibody adherence to its respective receptor and subsequently decreased serum availability [42].

Depression has a complex etiology, and its pathogenesis is not well-understood, but it has been proposed that immune dysregulation, due to autoantibody formation, could potentially play a role [43]. Studies on depression remain a challenge due to the lack of compatible animal models; however, strides have been made using autoantibodies to induce depression-like manifestations in murine models [44,45].

Several studies suggested that autoantibodies to NMDAR found in the hippocampus and the cortex neurons potentially increase the risk for depression [31,44,46]. A positive correlation between depressive disorders and serum autoantibodies to NMDAR, particularly to the NR2 subunit, exists in systemic lupus erythematosus (SLE) [47]. Moreover, neuropsychiatric manifestations in SLE patients are associated with ribosomal P proteins and endothelial-cell autoantibodies [48].

Endothelin-1 (ET1) and ET1 B-type receptors (ETBRs) signaling pathways in the amygdala have been shown to contribute to the attenuation of anxiety and depression [49] raising the possibility that receptor interference, through the presence of autoantibodies, would result in enhanced anxiety and depression. A recent study compared the clinical manifestations of fibromyalgia, depression, and ME/CFS in patients with SBIs and patients with SLE and scleroderma and concluded that fibromyalgia and ME/CFS is more common in patients with SBIs, compared with scleroderma controls [50].

Our current study highlights a cause–effect relationship between SBIs and diverse functional manifestations and also demonstrates the presence of certain autoantibodies to support such a relationship.

Our group recently showed that anti-β1AR might play a role in the development of autoimmune dysautonomia in symptomatic women with SBIs. Anti-β1AR was found to be significantly associated with autonomic-related nervous system manifestations such as sleep disturbances and depression [29]. The findings of this study support previous results and shows that circulating levels of anti-β1AR are significantly dysregulated in women with SBIs who suffered from sleep disturbance and depression (Figure 2 and Figure 4).

It is worth mentioning that our group and others found that SBI removal could improve functional symptoms such as cognitive impairment and depression, though it was not proven to benefit all SBI patients [51].

Preliminary data from in vitro studies conducted at our lab show that while total IgG secreted from lipopolysaccharide (LPS)-activated human monocytes derived from healthy subjects results in a reduction in pro-inflammatory cytokine (TNFα and IL-6) secretion, IgG derived from the blood of symptomatic SBI women increase the production of such cytokines. It remains to be tested whether the passive transfer of IgG autoantibodies derived from symptomatic SBI women into the brain of naïve mice will result in the appearance of these symptoms in murine models, thus proving a direct pathogenic effect of these autoantibodies.

Silicone implants serve as a classic example of how foreign material could act as an adjuvant in genetically predisposed individuals [52]. The seepage and migration of silicone into lymph nodes [10], and the engulfment of silicone microparticles by immune cells could result in hyperactivation of both innate and adaptive arms of the immune system. Such effects could explain the development of rare cases of T-cell lymphomas (BIA-ALCL) in women with SBIs and the production of classical and non-classical autoantibodies as a result of B-cell activation in other instances [5].

## 5. Limitations

Our study has a few limitations. The circulating levels of anti-GPPCRs autoantibodies were measured only once in symptomatic women with SBIs, preventing the detection of any fluctuations over time that could have correlated with the severity of symptoms. Medication history of symptomatic women with SBIs was not sought, which could have potentially influenced the circulating levels of anti-GPCRs antibodies. Symptoms were self-reported, and moving forward objective tests such as brain MRI/CT scans and routine cognitive screening tests are needed to validate, follow up, and explain the observed symptoms in affected patients.

Lastly, our study included a small sample size of women with SBIs, and therefore, further larger-scale studies are needed in order to further support our conclusions.

## 6. Conclusions

Female patients with SBIs have diverse clinical symptoms such as depression, cognitive impairment, and sleep disturbances. Such symptoms were found to be associated with dysregulated circulating levels of adrenergic, muscarinic, endothelin receptor type A, and type 1 angiotensin II receptor autoantibodies of the autonomous central nervous system. Autoantibodies against GPCRs of the autonomic nervous system might play significant roles in the development of suspected autoimmune dysautonomia-related disorders and might help explain some of the enigmatic, subjective CNS-related manifestations reported by patients.

## Figures and Tables

**Table 1:** Memory impairment.

Characteristic	Controls, *N* = 36 ^1^	Without Symptom, *N* = 40 ^1^	With Symptom, *N* = 53 ^1^
A1AR	15 (11, 20)	14 (11, 20)	13 (9, 17)
A2AR	12.2 (9.1, 14.5)	13.8 (9.7, 16.9)	11.5 (9.9, 14.8)
B1AR	23 (17, 44)	11 (8, 19)	9 (7, 13)
B2AR	6.9 (5.1, 11.5)	7.0 (4.3, 10.8)	6.8 (4.5, 10.0)
M1R	3.04 (2.28, 3.92)	3.09 (2.16, 4.38)	2.38 (1.83, 3.83)
M2R	5 (3, 8)	4 (3, 9)	3 (3, 5)
M3R	7.9 (6.4, 10.1)	6.8 (5.2, 9.7)	7.0 (5.3, 8.6)
M4R	9 (7, 12)	9 (6, 12)	6 (4, 9)
M5R	6.8 (5.3, 9.3)	7.7 (5.4, 10.8)	6.7 (5.4, 8.6)
AT1R	16 (12, 23)	11 (9, 17)	10 (8, 12)
ETAR	11.3 (9.4, 14.2)	9.3 (7.5, 12.3)	8.4 (6.9, 10.2)

^1^ Data presented as median (IQR) for the circulating titer of each antibody.

**Table 2:** Sleep disturbances.

Characteristic	Controls, *N* = 36 ^1^	Without Symptom, *N* = 41 ^1^	With Symptom, *N* = 52 ^1^
A1AR	15 (11, 20)	14 (11, 20)	12 (8, 16)
A2AR	12.2 (9.1, 14.5)	14.1 (11.5, 16.7)	10.9 (8.7, 15.5)
B1AR	23 (17, 44)	11 (9, 20)	9 (6, 12)
B2AR	6.9 (5.1, 11.5)	8.1 (5.3, 14.1)	5.8 (3.6, 9.3)
M1R	3.04 (2.28, 3.92)	3.12 (2.19, 4.34)	2.31 (1.49, 3.70)
M2R	5 (3, 8)	5 (3, 7)	3 (2, 4)
M3R	7.9 (6.4, 10.1)	7.7 (5.8, 8.9)	6.7 (4.9, 9.3)
M4R	9 (7, 12)	8 (7, 11)	5 (4, 10)
M5R	6.8 (5.3, 9.3)	7.5 (5.8, 11.2)	6.7 (5.1, 8.9)
AT1R	16 (12, 23)	11 (10, 14)	10 (8, 14)
ETAR	11.3 (9.4, 14.2)	9.3 (7.9, 11.8)	8.2 (6.3, 11.2)

^1^ Data presented as median (IQR) for the circulating titer of each antibody.

**Table 3:** Cognitive impairment.

Characteristic	Controls, *N* = 36 ^1^	Without Symptom, *N* = 51 ^1^	With Symptom, *N* = 42 ^1^
A1AR	15 (11, 20)	15 (11, 21)	12 (8, 15)
A2AR	12.2 (9.1, 14.5)	12.7 (10.5, 16.6)	11.5 (9.6, 15.9)
B1AR	23 (17, 44)	11 (8, 17)	9 (6, 11)
B2AR	6.9 (5.1, 11.5)	6.7 (4.4, 10.5)	7.0 (4.2, 10.3)
M1R	3.04 (2.28, 3.92)	2.58 (1.97, 4.22)	2.54 (1.81, 3.87)
M2R	5 (3, 8)	4 (3, 7)	3 (2, 5)
M3R	7.9 (6.4, 10.1)	7.1 (5.3, 9.6)	6.8 (5.0, 8.7)
M4R	9 (7, 12)	8 (6, 12)	5 (4, 8)
M5R	6.8 (5.3, 9.3)	7.6 (5.6, 11.6)	6.7 (4.9, 8.3)
AT1R	16 (12, 23)	11 (9, 16)	10 (8, 13)
ETAR	11.3 (9.4, 14.2)	9.2 (7.8, 12.0)	8.1 (6.6, 10.6)

^1^ Data presented as median (IQR) for the circulating titer of each antibody.

**Table 4:** Depression.

Characteristic	Controls, *N* = 36 ^1^	Without Symptom, *N* = 56 ^1^	With Symptom, *N* = 37 ^1^
A1AR	15 (11, 20)	14 (11, 19)	11 (8, 16)
A2AR	12.2 (9.1, 14.5)	14.2 (10.9, 16.9)	10.3 (8.4, 12.4)
B1AR	23 (17, 44)	11 (8, 21)	8 (6, 11)
B2AR	6.9 (5.1, 11.5)	7.5 (4.9, 10.8)	5.1 (3.5, 8.4)
M1R	3.04 (2.28, 3.92)	3.05 (2.21, 4.37)	2.13 (1.51, 3.67)
M2R	5 (3, 8)	4 (3, 8)	3 (2, 4)
M3R	7.9 (6.4, 10.1)	7.7 (5.7, 9.7)	5.9 (4.9, 8.1)
M4R	9 (7, 12)	9 (6, 11)	5 (4, 7)
M5R	6.8 (5.3, 9.3)	7.6 (5.8, 11.7)	6.3 (4.9, 8.2)
AT1R	16 (12, 23)	11 (9, 16)	10 (8, 11)
ETAR	11.3 (9.4, 14.2)	9.3 (8.0, 12.1)	7.7 (6.2, 9.7)

^1^ Data presented as median (IQR) for the circulating titer of each antibody.

## Data Availability

All data and materials, as well as software applications, support our published claims and comply with field standards.
